# Outcomes of nonrejection in weakly fluorescent intestine detected by indocyanine green fluorescence angiography: a case series of infants

**DOI:** 10.1186/s40792-024-01885-y

**Published:** 2024-04-24

**Authors:** Naoki Hashizume, Akihiro Yoneda, Genta Ozeki, Takeshi Saito, Michimasa Fujiogi, Motohiro Kano, Yuki Yamamoto, Tetsuya Ishimaru, Yutaka Kanamori, Akihiro Fujino

**Affiliations:** 1 Division of Surgery, Department of Pediatric Surgical Specialties, National Center for Child Health and Development, 2-10-1 Okura, Setagaya-ku, Tokyo 157-8535 Japan; 2 Department of Pediatric Surgery, Kurume University School of Medicine, 67 Asahimachi, Kurume, Fukuoka 830-0011 Japan; 3 Division of Surgical Oncology, Children’s Cancer Center, National Center for Child Health and Development, 2-10-1 Okura, Setagaya-ku, Tokyo 157-8535 Japan; 4 Department of Pediatric Surgery, Keio University, 35 Shinanomachi, Shinjuku-ku, Tokyo 160-8582 Japan

**Keywords:** Indocyanine green, Fluorescence angiography, Intestinal perfusion, Necrotizing enterocolitis, Infant

## Abstract

**Background:**

Indocyanine green fluorescence angiography, a validated noninvasive imaging technique, is used to assess tissue vascularization. Here, we report three infant patients who underwent intraoperative indocyanine green fluorescence angiography and suffered from postoperative complications caused by the lack of weak fluorescent intestinal resection and assessed residual intestinal perfusion.

**Case presentation:**

We observed the clinical characteristics and operative findings of patients treated from January 2022 to December 2022. Indocyanine green (0.5 mg/kg) was intravenously injected. The first patient was a 29-day-old girl with surgical necrotizing enterocolitis who underwent intraoperative indocyanine green fluorescence angiography at the first- and second-look operations. The proximal jejunum was difficult to diagnose to detect blood flow during the second-look operation. The second patient was a 32-day-old boy with surgical necrotizing enterocolitis. A part of the antimesenteric mucosa of the patient that exhibited weak fluorescence was preserved; however, it formed postoperative hematomas. The third patient was a 30-day-old boy with midgut volvulus. Weak fluorescence in the intestinal wall was observed 5 cm of the small intestine from the ileocecal valve was preserved, but it formed a stricture, and the patient underwent ileocecal resection after 30 days.

**Conclusions:**

Weak fluorescence in the intestine in infants by performing indocyanine green fluorescence angiography is associated with a high risk of non-recovering ischemic lesions and postoperative complications.

**Supplementary Information:**

The online version contains supplementary material available at 10.1186/s40792-024-01885-y.

## Introduction

Intestinal ischemia for midgut volvulus and/or necrotizing enterocolitis (NEC) in infants is life-threatening, and massive bowel reduction results in short-bowel syndrome. However, intraoperative evaluation of the clinical findings of the vascular ischemic intestine, such as serosal surface color, pulsation, and bleeding from the marginal arteries, has been based on the subjective evaluation of the operative surgeon [1]. To avoid massive bowel reduction for irrational reasons, leaving an ischemic bowel results in anatomical leakage and bowel stricture. Recently, indocyanine green fluorescence angiography (ICG-FA), a validated noninvasive imaging technique, has been used to evaluate tissue vascularization and guide intraoperative decisions in many surgical fields, including plastic surgery, neurosurgery, and general surgery [2, 3]. ICG, a fluorescent agent, is a water-soluble dye with peak spectral absorption and emission at 800–810 nm in the blood or plasma. Recently, ICG-FA was reported to be a safe and crucial imaging modality in the pediatric population without side effects [1, 2]. However, few cases underwent blood perfusion measurement of the intestine in the neonatal period using ICG-FA [4]. Moreover, none of the cases underwent blood perfusion measurement after midgut volvulus and the decision of no resection of the weak fluorescent intestine in the infant period.

Here, we report three cases of infant patients who underwent intraoperative ICG-FA to assess residual intestinal perfusion and suffered from postoperative complications caused by the lack of weak fluorescent intestinal resection.

## Methods

The clinical presentations and operative findings of two patients with surgical NEC and one patient with midgut volvulus treated from January 2022 to December 2022 are presented in Table 1. In all cases, the ICG fluorescence device used a photodynamic eye fluorescence system: (PDE-neo®) (Hamamatsu Photonics, Japan), which is a hand-held camera featuring manual adjustment, including an infrared excitation wavelength of 760 nm, which collects 830 nm wavelength fluorescence emitted by ICG and visualizes it on a monitor in real time [5]. ICG of 0.5 mg/kg was injected intravenously.

**Table 1:** Patients characteristics

Case	Past medical history	Age at operation (day)	Operative findings	Fluorescent findings after injection			Decision	Formation of intestine after operation
				Intestine	120 s	600 s		
1	Meconium-related ileus, post ileostomy	29	NEC	Total small intestine	No	Weak	Re-evaluate after 24 h	Ischemic bowel recovered; but a defection in the mucosa on the antimesenteric side of the proximal jejunum
		30		The proximal jejunum	Weak	Weak	No resection	Stricture
2	None	32	NEC	–	–	–	A silo and ileostomy/re-evaluation	Ischemic bowel recovered
		33		60-cm length of jejunum–ileum	No	No	Resection	–
				A part of the anti-mesenteric mucosa of jejunum	Weak	Weak	No resection	Hematomas in the mucosa
3	7q distal trisomy syndrome, ventricular septal defect	30	Midgut volvulus	Intestinal wall observed 5 cm of the small intestine from the ileocecal valve	Weak	Weak	No resection	Stricture

*NEC* necrotizing enterocolitis

## Cases

### Case 1

A female child was delivered at 32 weeks of gestation with a body weight of 1300 g. She underwent ileostomy after 6 days of birth because of meconium-related ileus. The operative findings indicated no intestinal malrotation.

**Fig. 1:**
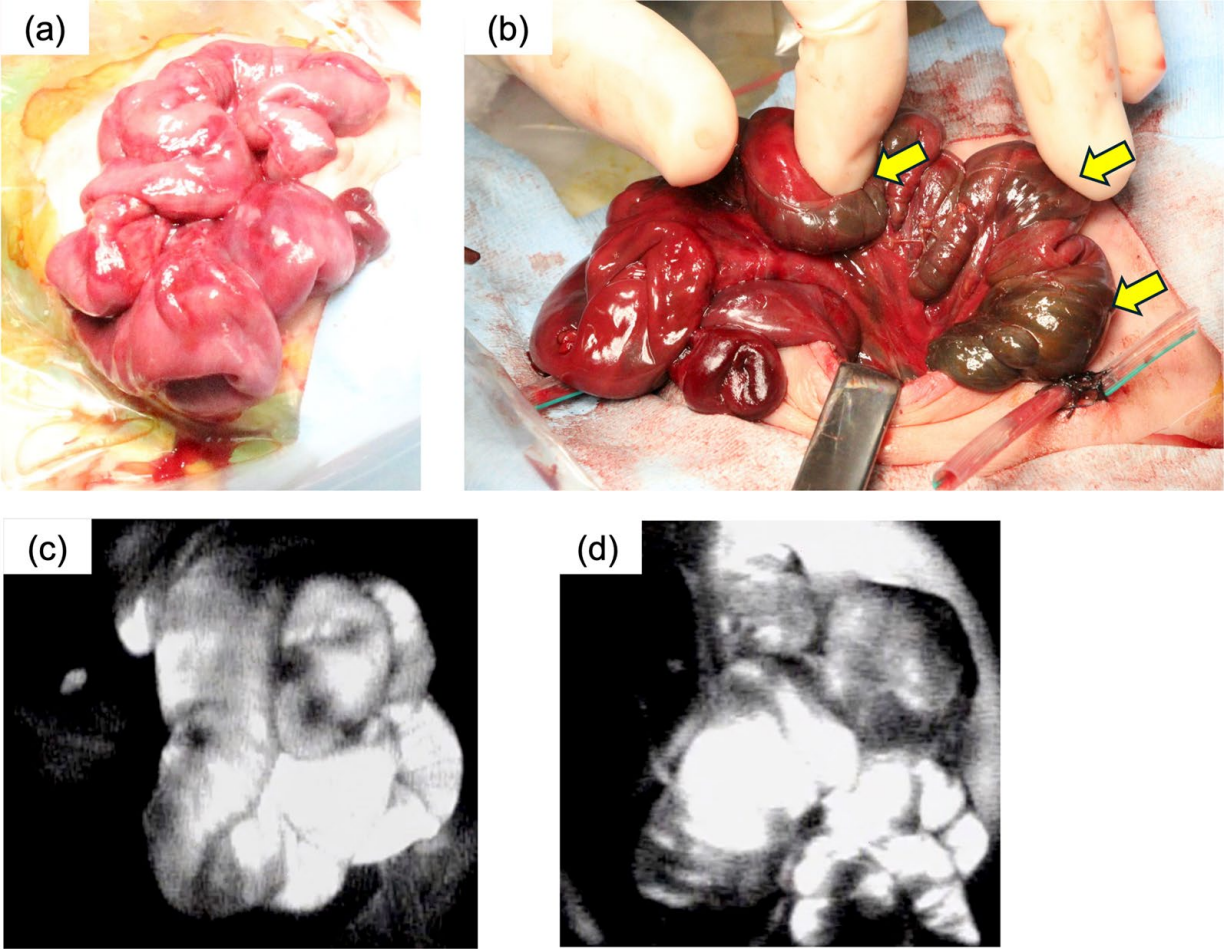
Case 1: **a** Operative findings show that almost the entire small bowel was inflamed. **b** Operative findings at the second-look operation revealing that the remaining ischemic bowel recovered, but the proximal jejunum demonstrated a defect in the mucosa on the antimesenteric side (arrows). **c** Before ICG injection, the small intestine fluoresced because of ICG-FA from the first operation. **d** The proximal jejunum exhibited weak fluorescence 600 s after ICG injection (arrows)

The patient developed persistent abdominal distension and bloody stool at 29 days of age. An abdominal radiograph revealed ascites; however, it could not detect superior mesenterial artery location for intestinal gas bloating. The patient was suspected of having NEC. Her condition rapidly deteriorated with marked metabolic acidosis worsening. An emergency laparotomy was performed after the patient recovered from shock. The majority of the small bowel was inflamed during laparotomy (Fig. 1a). ICG-FA revealed no fluorescence in the entire strangulated intestine until 120 s of ICG injection. Dark-colored and weak fluorescence was observed in the intact mesentery and bowel wall after 600 s. Hence, we decided to reevaluate the viability of the small intestine without resection (Additional file 1: Video S1).

A second-look operation was performed after 24 h of laparotomy. Although the remaining ischemic bowel recovered, the proximal jejunum demonstrated a defect in the mucosa on the antimesenteric side (Fig. 1b). The small intestine showed fluorescence before ICG injection because of ICG-FA from the first operation (Fig. 1c); therefore, we decided to reperform ICG-FA. The proximal jejunum was difficult to diagnose after 600 s of ICG injection at ICG-FA (Fig. 1d). The patient was at risk for ultrashort bowel syndrome with massive intestinal resection due to the proximal jejunum; therefore, this section was not resected and a jejunostomy was created.

However, improvement was poor, and widespread small intestinal necrosis occurred in the proximal jejunum. Small intestinal resection was performed at 85 days of age, and retrograde tube duodenostomy was performed. The patient died at 97 days of age.

### Case 2

**Fig. 2:**
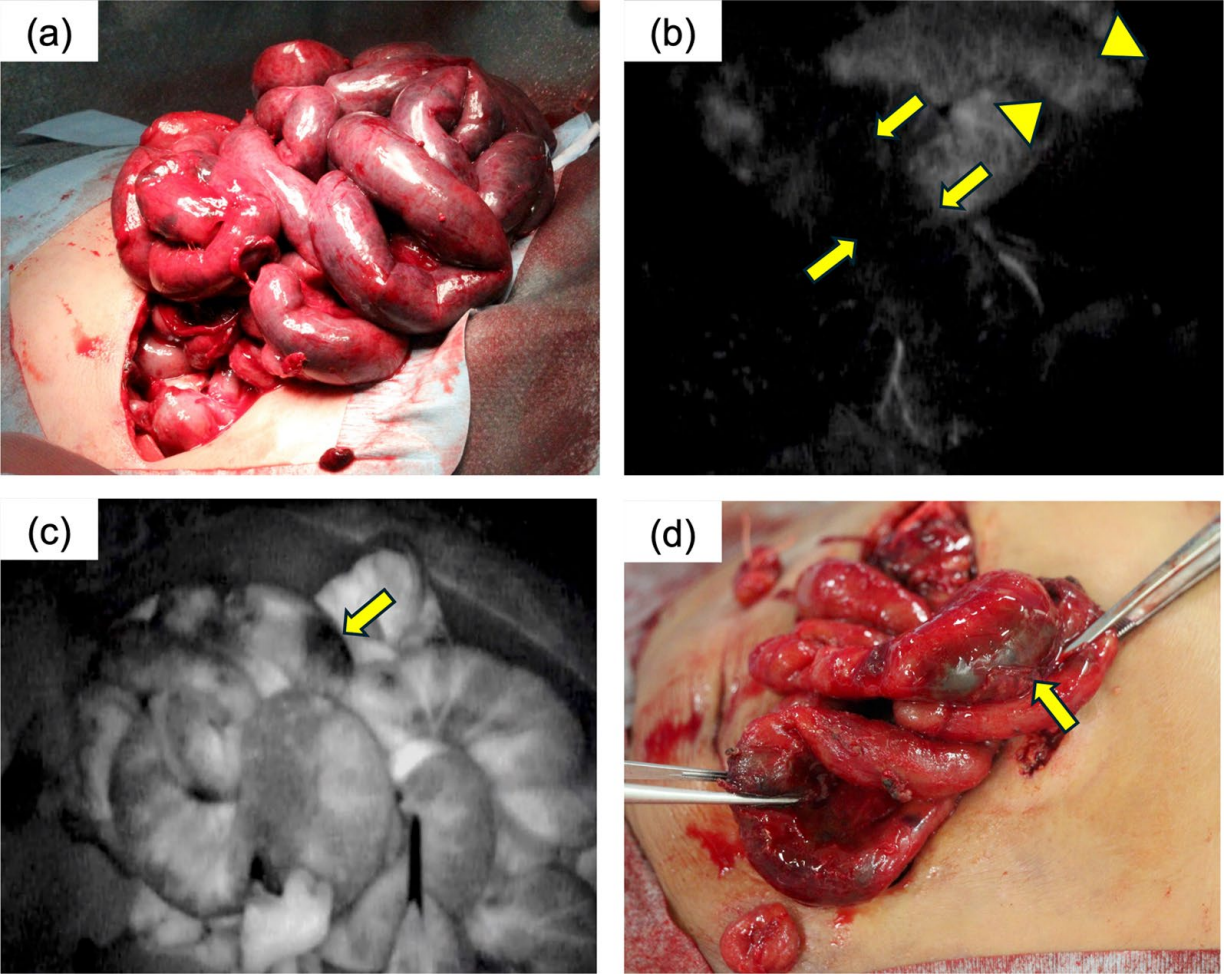
Case 2: **a** Operative findings reveal widespread ischemic changes in the small intestine. **b** Operative findings at the second-look operation by ICG-FA showing that a 60-cm length of the jejunum–ileum did not fluoresce after 600 s after ICG injection (arrows). The proximal jejunum demonstrated mild fluorescence (arrowheads). **c** A part of the antimesenteric mucosa exhibited weak fluorescence (arrow). **d** At 60 days of age, surgical findings reveal the formation of hematomas in the mucosa causing stenosis (arrow). It was resected

A male baby was delivered at 42 weeks of gestation with a body weight of 3002 g. The patient experienced sudden vomiting at 32 days of age, and his condition rapidly deteriorated, marked by worsening metabolic acidosis and acute kidney failure. Observations revealed abdominal distention, ascites, and circulatory failure, which indicated an increased possibility of abdominal compartment syndrome. An exploratory laparotomy was performed that revealed widespread ischemic changes in the small intestine (Fig. 2a). A silo and ileostomy were created 15 cm from the proximal jejunum. Subsequently, the viability of the small intestine was re-evaluated.

A second-look operation was conducted after 24 h of initial laparotomy. The remaining ischemic bowel was recovered. Fluorescence was observed during ICG-FA in the 30-cm proximal jejunum until 30 s after ICG injection. The jejunum demonstrated fluorescence in the mesentery and intestinal wall until 60–120 s after ICG injection. Entire intestine, excluding a 60-cm length of the jejunum–ileum, exhibited fluorescence in the intact mesentery and bowel wall by ICG-FA after 600 s (Fig. 2b, Additional file 2: Video S2). Nonfluorescent intestine was resected, and a separated ileostomy was performed. In addition, a part of the antimesenteric mucosa of the jejunum revealed weak fluorescence during ICG-FA, but it was preserved (Fig. 2c). However, the mucosa demonstrated poor passage after enteral nutrition feeding postoperatively.

The formation of hematomas in the mucosa was observed during laparotomy at 60 days of age, and the stoma was closed (Fig. 2d).

### Case 3

**Fig. 3:**
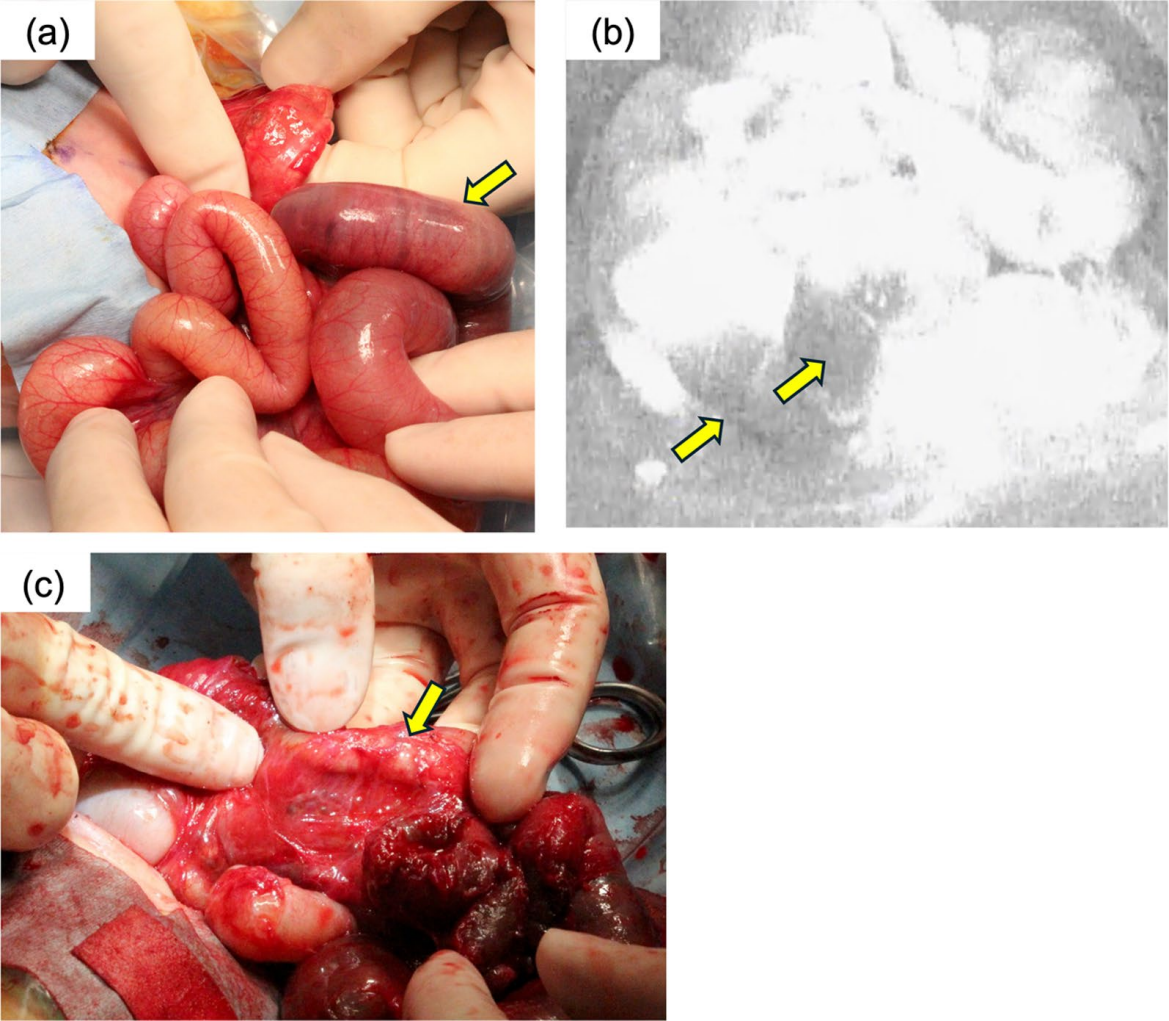
Case 3: **a** operative findings revealing recovering ischemic changes in the small intestine (arrow). **b** Operative findings by ICG-FA showing that 5 cm of the ileum demonstrated weak fluorescence (arrow). **c** At the age of 61 days, the surgical findings show the formation of a stricture (arrow)

A male child was delivered at 38 weeks of gestation with a body weight of 2522 g. He demonstrated 7q distal trisomy syndrome and ventricular septal defect and underwent abdominal detention and enema at 30 days of age. Abdominal radiological results revealed portal vein gas occurrence. The midgut volvulus required emergency laparotomy. Operative results revealed an ileum and ascending colon volvulus with malrotation that rotated in a reverse clockwise direction of 180°. Detorsion was performed. Almost the entire small bowel, except 5 cm from the ileocecal valve, showed strong fluorescence in the mesentery and intestinal wall until 90 s after ICG injection (Fig. 3a). The 5-cm small intestine located from the ileocecal valve demonstrated weak fluorescence in the intestinal wall, but the mesentery was fluorescent after 600 s (Fig. 3b, Additional file 3: Video S3). This area was preserved. However, bowel dilatation remained after initiating enteral feeding. This area formed a stricture, and ileocecal resection was performed during stoma closure after 60 days of age (Fig. 3c).

## Discussion

To the best of our knowledge, there were few reports to show the preservation of the intestine by weak fluorescence using ICG-FA for predicting the viability of ischemic small bowel in infants [6]. Although this method is effective for visually evaluating blood flow, it remains difficult to determine its reliability because there is no objective index that can be quantified, such as the reversible fluorescence onset time of the ischemic intestinal tract or its amount of fluorescence.

Matsui et al. [7] revealed that intraoperative ICG-FA was more accurate in predicting the survival from ischemic bowel than clinical evaluation alone with qualitative and quantitative ICG-FA. The risk factors for anastomotic leakage were mainly calculated using the slope of fluorescence intensity, background-subtracted peak fluorescence intensity, and peak time from fluorescence. They classified the vascular flow patterns of the ischemic bowel using ICG-FA into four groups: (1) normal, (2) delayed venous drainage, (3) capillary insufficiency, and (4) arterial insufficiency. Based on this classification, we considered that, the pattern of the changes in the ICG fluorescence intensity of the intestine at first-look operation findings corresponded to the arterial insufficiency pattern and at second-look operation findings to the delayed drainage pattern in case 1. In the other two cases, the ICG fluorescence intensity pattern of the intestine changes during the operation corresponded to the arterial insufficiency pattern. A strong correlation was observed between regional blood flow and the slope of the fluorescence curves (Intensity/Δtime) when stomach tissue samples from pigs obtained from the sites of interest were sent for microsphere quantification to calculate regional blood flow and ICG-FA [8]. Iinuma et al. revealed that changes in fluorescence intensity over time were measured using an image intensity analysis software after 14 years of midgut volvulus diagnosis. The fluorescence intensity at the mesentery gradually increased over time, whereas that observed at the distal part of the residual jejunum demonstrated only a persistently slow increase in intensity and the absence of gradual increases over time [9]. The ischemic intestinal tract becomes slowly and progressively fluorescent despite the gradually increasing mesentery, as observed in our cases. These findings were diagnosed as a capillary insufficiency pattern and may be a risk factor for non-reserving ischemic conditions. However, quantification of ICG-FA in a human model of the ischemic intestine has not reached consensus.

For another outcome of ICG-FA, a cutoff protocol for the fluorescence onset time is required. According to a systematic review in 2022 in 15 studies for reduction rate of anastomotic leakage in rectal cancer surgery in adult, the fluorescence onset time was 60 s in 13 studies, 30 s in 1 study, 90 s in 1 study, and 2 to 3 min in 1 study. In most studies, the fluorescence time before resection occurred was 60 s. Shirota et al. [10] reported the cutoff for blood flow in the intestine protocol “within 70 s after ICG administration” based on gastric tube reconstruction for adult patients and did not report any cases of postoperative anastomotic stenosis or leakage [6]. In our cases, a part of the ischemic intestine was recovered otherwise, the intestine in which the fluorescence onset time was over 120 s. The fluorescence onset time for preserving the intestine was > 120 s, considering the recovery of vitality, intestinal decompression, and acidosis for NEC and volvulus. However, after 600 s of injection, the fluorescence of weakly ischemic intestines, especially part of the antimesenteric area, was not recovered.

The small intestine remained fluorescent because of ICG-FA from the first operation before 24 h in case 1 during the second-look operation. Patients with NEC and midgut volvulus underwent a second-look operation to avoid massive bowel reduction. ICG was excreted in bile and restored in the intestine, which made diagnosing fluorescence intensity difficult during the second-look operation. ICG-FA was recommended to not be used at the first operation if the second operation was planned for a more accurate fluorescence pattern.

Regarding the surgical dosage, 0.5 mg/kg of ICG is sufficient for testing intraoperative decision-making in lung metastasis of hepatoblastoma [5]. Another study revealed that patients receiving ICG to visualize biliary flow were administered ICG by intravenous injection at 0.05–0.5 mg/kg [2, 4, 6]. Breuking et al. reviewed the ICG safety for use in neonates because all studies except one reported the absence of any complication or adverse event occurring when ICG is used at the dosage in infants aged ≤ 3 months. Side effects of retention of the injected dye or change of skin color after ICG administration are temporary and fully disappear in several weeks [2]. No data indicated a difference in intensity by the dosage of ICG in humans. Thus, further studies are required.

## Conclusion

Preserving weak fluorescence in the intestine in infants by ICG-FA is associated with a high risk of non-recovering ischemic lesions and postoperative complications. Prospective further studies with larger sample sizes are required to elucidate the relationship between postoperative intestinal function and vascular flow patterns of ICG-FA in infants.

### Supplementary Information

**Additional file 1: Video S1.** Intra-operative ICG-FA of case 1.

**Additional file 2: Video S2.** Intra-operative ICG-FA of case 2.

**Additional file 3: Video S3.** Intra-operative ICG-FA of case 3.

## Data Availability

All data generated during this study are included in this published article.

## Abbreviations

ICG: Indocyanine green
ICG-FA: Indocyanine green fluorescence angiography
NEC: Necrotizing enterocolitis
